# Serum calcium and magnesium in preeclampsia: A comparative study

**DOI:** 10.6026/973206300214222

**Published:** 2025-11-15

**Authors:** Himanshy Rai, Shweta Yadav, Shambhavi Soni, Pratiksha Yadav, Meghna Singh Jadon, Mayank Pratap Singh

**Affiliations:** 1 Department of Obstetrics and Gynaecology, Government Medical College, Datia, Madhya Pradesh, India; 2 Department of Public Health, Datta Meghe Institute of Higher Education and Research Deemed to be University, Wardha, Maharashtra, India

**Keywords:** Preeclampsia, serum calcium, serum magnesium, pregnancy-induced hypertension, maternal outcomes, fetal outcomes

## Abstract

Preeclampsia is a hypertensive disorder of pregnancy linked to endothelial dysfunction and electrolyte imbalance. Therefore, it is of interest to evaluate serum calcium and magnesium levels in 100 preeclamptic and 100 normotensive pregnant women beyond 28 weeks gestation at a tertiary hospital in Central India. Spectrophotometric analysis showed significantly lower calcium (7.84 ±0.74 mg/dL vs. 9.68 ±0.97 mg/dL; p<0.001) and magnesium levels (1.74 ±0.54 mg/dL vs. 1.88 ±0.76 mg/dL; p=0.040) in preeclamptic women. Adverse outcomes such as preterm birth, cesarean delivery, and NICU admissions were more frequent in the preeclampsia group. These findings suggest the utility of calcium and magnesium screening in antenatal care for early risk stratification and potential prevention of complications.

## Background:

Preeclampsia is a severe multi-organ disorder that typically manifests after 20 weeks of pregnancy, most often in the third trimester, and is characterized by shallow cytotrophoblast invasion and inadequate vascular remodeling, leading to placental hypoperfusion and systemic endothelial dysfunction, which results in vasoconstriction, hypoxia, and organ involvement, often exacerbated by comorbidities such as diabetes, obesity, and chronic inflammation [1]. Molecular mediators such as endothelin-1, soluble fms-like tyrosine kinase-1 (sFlt-1), angiotensin II type 1 receptor autoantibodies (AT1-AA), and reduced nitric oxide levels have been implicated in its pathogenesis [2]. Altered mineral metabolism has been associated with preeclampsia, particularly reduced serum calcium and magnesium levels, and a study identified a calcium-magnesium ratio cutoff of 2.36 as a potential risk threshold for the disorder [3]. A Nigerian study confirmed significantly lower serum calcium and calcium-magnesium ratios in affected women, underscoring calcium's role in vascular tone and blood pressure regulation [4]. A case-control study in Ethiopia linked low dietary calcium intake and reduced serum calcium (both total and ionized) to a higher risk of developing preeclampsia [5]. A study examining women treated with magnesium sulfate found a direct association between higher magnesium levels and increased maternal and perinatal mortality [6]. Elevated sFlt-1/PlGF ratios correlate with adverse maternal and fetal outcomes [7]. Dysregulation of these markers in women with chronic hypertension in the first trimester has shown predictive value for later development of preeclampsia [8]. Therefore, it is of interest to eualuvate serum calcium and magnesium in preeclampsia.

## Materials and Methods:

This cross-sectional study was conducted to evaluate serum calcium and magnesium levels in pregnant women with and without preeclampsia. It was carried out in the Department of Obstetrics and Gynecology at a tertiary care hospital in Central India over a period of eight months, from 1 September 2024 to 30 April 2025. A total of 200 pregnant women were enrolled, comprising 100 preeclamptic cases and 100 normotensive controls. Participants were selected using systematic random sampling from both outpatient and inpatient obstetric units. Inclusion criteria were: age between 18 and 35 years, singleton pregnancy, gestational age above 28 weeks, and parity less than five. Preeclampsia was defined as blood pressure ≥140/90 mmHg on two separate occasions at least four hours apart, along with proteinuria >300 mg/24 hours or a dipstick reading of +1 or more. Normotensive controls were matched for age and gestational age and had blood pressure <140/90 mmHg with no proteinuria. Ethical approval was obtained from the institutional ethics committee, and written informed consent was collected from all participants. Women with essential hypertension, asthma, hematological disorders, cardiovascular disease, severe hepatic or renal impairment, multiple gestations, or hemodynamic instability were excluded. Detailed medical and family histories were recorded, followed by thorough clinical examinations. Blood pressure was measured using a standard sphygmomanometer placed one inch above the cubital fossa, with the arm at heart level in a seated position. The diastolic pressure was determined using the fifth Korotkoff sound (K5), measured twice, four hours apart, following a minimum rest of 30 minutes. Proteinuria was assessed using a clean-catch midstream urine sample, tested by dipstick, and graded from 0 to +4. Peripheral venous blood was drawn from the cubital vein and analyzed for serum calcium and magnesium. Calcium was estimated using the spectrophotometric method with the Beckman Coulter X2-5 Automatic Analyzer and CA2 300 calcium reagent. Magnesium was measured using the Xylidyl Blue method on the Roche Hitachi 902 analyzer. Samples were stored at -20°C when immediate analysis was not feasible. Participants were followed until delivery, and fetal outcomes were documented. Data collection was performed using a pre-structured, pre-tested proforma. Statistical analysis was conducted using Microsoft Excel and Jamovi software (v2.3.28). Frequencies, proportions, and measures of central tendency were calculated. Appropriate statistical tests were applied, and a p-value of <0.05 was considered statistically significant.

## Results:

This study analyzed demographic, clinical, and biochemical parameters in 200 pregnant women, including 100 preeclamptic cases and 100 normotensive controls. No significant differences were found in age, residential area, or socioeconomic status (p > 0.05). BMI was significantly higher in the preeclampsia group (p < 0.001) (Table 1 - see PDF). Systolic and diastolic blood pressures were significantly higher in the study group (p < 0.001). Hemoglobin and platelet counts were lower, while uric acid levels were elevated in preeclamptic women (p < 0.001). Only 25% of preeclamptic women reported calcium supplementation, compared to 45% of controls (p = 0.003) (Table 2 - see PDF). Preterm delivery (<37 weeks) was more frequent in cases (78.0% vs. 56.0%, p < 0.001), and cesarean sections were more common (22.0% vs. 8.0%, p = 0.007). NICU admissions (40.0% vs. 10.0%, p < 0.001) and neonatal deaths (8.0% vs. 1.0%, p = 0.004) were also significantly higher in the study group. APGAR scores at 5 minutes and birth weights were lower in preeclamptic women (p = 0.001) (Table 2 - see PDF). Serum calcium levels were significantly lower in preeclamptic women (7.84 ± 0.74 mg/dL vs. 9.68 ± 0.97 mg/dL, p < 0.001), and serum magnesium levels were also reduced (1.74 ± 0.54 mg/dL vs. 1.88 ± 0.76 mg/dL, p = 0.040) (Table 3 - see PDF).

## Discussion:

The lack of differences in age, residence, or socioeconomic status between groups suggests these factors did not confound the observed disparities. Higher BMI in preeclamptic women aligns with established evidence linking obesity to hypertensive pregnancy disorders [1]. Elevated blood pressures, lower hemoglobin and platelet counts, and raised uric acid levels reflect the known pathophysiology of preeclampsia. Significantly lower calcium supplementation in preeclamptic women supports findings from Ethiopia associating low dietary calcium and serum calcium with increased preeclampsia risk [5], reinforcing WHO's supplementation guidelines. Our observation of reduced serum magnesium mirrors results by Wadhwani *et al.* who identified low mid-pregnancy magnesium as a predictor of preeclampsia [9], and by Tesfa *et al.* who confirmed this association in African women [10]. These findings suggest hypomagnesemia may contribute to disease via endothelial dysfunction and inflammation. Increased preterm deliveries, cesarean rates, NICU admissions, and neonatal deaths in the preeclampsia group reflect adverse outcomes linked to metabolic imbalances [1, 10]. Zhang *et al.* similarly noted that adequate magnesium intake may reduce preterm birth risk [11]. Although Araújo *et al.* found magnesium supplementation ineffective in low-risk women; its safety supports targeted use in high-risk groups [12].

## Conclusion:

Deficiencies in calcium and magnesium are significantly linked to adverse maternal and fetal outcomes, emphasizing their importance in maternal health. Routine screening and timely supplementation of these electrolytes during antenatal care could help prevent hypertensive disorders in pregnancy and improve clinical outcomes. Further studies should explore combining these biomarkers with other predictive indicators to enhance early detection and management of preeclampsia.
